# Exploring the effect of ritonavir and TMC-310911 on SARS-CoV-2 and SARS-CoV main proteases: potential from a molecular perspective

**DOI:** 10.2144/fsoa-2020-0079

**Published:** 2020-11-09

**Authors:** Opeyemi S Soremekun, Kehinde F Omolabi, Adeniyi T Adewumi, Mahmoud ES Soliman

**Affiliations:** 1 Molecular Bio-computation & Drug Design Laboratory, School of Health Sciences, University of KwaZulu-Natal, Westville Campus, Durban 4001, Kwa-Zulu Natal, South Africa

**Keywords:** COVID-19, drug repurposing, ritonavir, SARS-CoV, SARS-CoV-2, TMC-310911

## Abstract

**Aim:**

As coronavirus (CoV) disease 2019-associated pneumonia spreads globally, there has been an urgent need to combat the spread and develop vaccines.

**Materials & methods:**

We used an integrated computational algorithm to explore the binding mechanism of TMC-310911/ritonavir (RVT) with SARS-CoV-2 and SARS-CoV main proteases.

**Results:**

RVT and TMC-310911 had favorable interactions with the proteases, and these high interactions are facilitated by some significant residues such as Asn133, Gly195 and Gln192. Our study further implicated two important rings in the structure of RVT as a possible chemical culprit in its therapeutic activity.

**Conclusion:**

Although there are conflicting clinical results on the therapeutic potency of RVT in the treatment of coronavirus disease 2019, our findings provided molecular insight into the binding mechanism of TMC-310911 and RVT with SARS-CoV-2 and SARS-CoV main proteases.

## Lay abstract

As coronavirus (CoV) disease 2019 spreads, there is an urgent need to develop drugs that can halt the spread, manage and perhaps cure this disease. We repurposed two antiretroviral drugs that target the same protein as SARS-CoV-2 (the virus that causes coronavirus disease 2019). Our findings revealed that these two drugs demonstrated favorable activity on SARS-CoV-2 protein.

SARS coronavirus 2 (SARS-CoV-2) has been implicated as the causative agent for the recent global pandemic disease named CoV disease 2019 (COVID-19). COVID-19 disease originates from the *Coronaviridae* group [1]. SARS-CoV-2 is related to the SARS-CoV [2], which caused a pandemic in 2003 particularly in Asia, and the Middle East respiratory syndrome-related CoV (MERS-CoV) [3]. These viruses have a positive sense, ssRNA and close structural similarities [4]. In comparison with the other CoVs, there are 380 substituted amino acids in SARS-CoV-2 [5]. Moreover, the phylogenetic analyses of the whole genome demonstrated that the SARS-CoV-2 protease was closely related to the bat-like SARS-CoV protease, but was distinct from the MERS-CoV protease [5]. In comparison, while the 8a protein is present in SARS-CoV, it is absent in SARS-CoV-2. The 8a proteins have been discovered to induce apoptosis and also facilitate viral replication [6]. Furthermore, the previous genome sequence demonstrated that 8b and 3b SARS-CoV-2 proteins containing 84 and 154 amino acid residues, have 121 and 22 amino acids in the human SARS-CoV, respectively [5]. COVID-19 is thought to be significantly less deadly when compared with the mortality rate of SARS (∼10%) [7] or MERS [7]; unfortunately, the reproductive number of COVID-19 is higher than that of MERS and SARS [8], therefore, its mortality rate if not controlled early is estimated to surpass that of SARS and MERS. According to the WHO (Geneva, Switzerland), as of 7 June 2020, there are 6,799,713 cases and 397,388 deaths globally [9]. Reports indicate that there is a cross-transmission of CoVs infections across different species including humans [10]. The concerns of the global populace, including in the scientific community, is the exponential increase in the number of infected persons. Some treatment regimens have been reported to have helped patients overcome the infection through boosting their immune system [8]; however, currently, no vaccines or drugs are available for the treatment of this disease [11]. The global spread of COVID-19 and the absence of vaccines or drugs have necessitated the demand of drug repurposing. Crucial proteins that have gained attention in the development of potential COVID-19 drugs include SARS-CoV-2 main proteases (COV), RNA-dependent RNA polymerase, RNA binding N terminal domain of nucleocapsid protein, viral ion channel, 2′O-ribosemethyltransferase and human angiotensin-converting enzyme 2 receptor [12–14]. Several existing drugs such as remdesivir, chloroquine, hydroxychloroquine, camostatmesylate, lopinavir, etc., have been considered for targeting different phases of the virus life cycle.

Ritonavir (RVT) is an antiretroviral drug used in the treatment of advanced HIV, it is used as a protease inhibitor and also used to boost other protease inhibitors [15]. It is sometimes used in combination with antiretroviral drugs such as nucleotide reverse transcriptase inhibitor or nucleoside [15,16]. An *in vivo* study found a reduced risk of mortality and hypoxia in 41 SARS-CoV patients who were administered a combination of lopinavir/RVT and ribavirin, when compared with controls administered ribavirin alone [17,18]. A different study from Korea revealed that there was a significant reduction in CoV titers upon lopinavir-RVT administration [19]. Contradictory, a combination of lopinavir and RVT was investigated in a controlled trial study, patients with COVID-19 were administered either lopinavir-RVT 400/100 mg orally twice daily plus standard of care, or standard of care alone, there was no therapeutic benefit observed upon administration [20]. There are at least three randomized clinical trials currently been carried out to determine the therapeutic efficacy of a combination of lopinavir and RVT [21]. Similarly, ASC-09 also referred to as TMC-310911 is a protease inhibitor that is still under clinical studies to treat HIV infection. It has shown *in vitro* activity against the HIV strains that have developed resistance to other protease inhibitors [22]. There is currently an ongoing clinical trial that seeks to evaluate the safety and efficiency of a combination of ASC09/RVT on CoV infection (clinicaltrials.gov/ct2/show/NCT04261907) [23].

In this study, we repurposed two existing HIV-protease inhibitors RVT and TMC-310911 (ASC09) to target SARS-CoV-2 main protease (COV) and SARS-CoV main protease (SARS) to explore their possible mode of inhibition.

## Structure preparation & dynamic studies

The starting structures of COV and SARS were obtained from Protein Data Bank with PDB ID 6LU7 [24] and 5N19 [25], respectively. Cocrystalized molecules identified with the proteins were deleted and the addition of missing residues was performed using modeler [26]. RVT and TMC-310911 were retrieved from PubChem, the 2D structures were converted to 3D structures and optimized using B3LYP/6-311++G(d,p) [27] level of Gaussian16 [28]. The final structures were saved as Mol2 files. Molecular docking of the proteases with the inhibitors was carried out using AutoDock Vina [29] inbuilt in UCSF chimera [30]. AutoDock Vina is software developed by O Trott of Molecular Graphics Laboratory at the Scripps Research Institute [29]. Validation of the docking results was carried out by redocking multiple times. COV and SARS were prepared for docking by the removal of water and cocrystallized ligands. Hydrogens were added and optimization of the hydrogen-bonding network was carried out with the aid of Avogadro software [31]. The clean COV and SARS structures were saved for docking. During the docking process, we allotted partial chargers called Gasteiger to the ligands. The AutoDock [32] GUI provided by the Molecular Graphics Laboratory tool was utilized to define the AutoDock atomic types [33]. The defined parameter grid dimensions used in docking TMC-310911 with COV and SARS were center (x = -17.56, Y = 0.29 and Z = -21.46) and size (X = 16.00, Y = 16.06 and Z = 13.19), while center (x = -17.19, Y = 0.44 and Z = -21.95) and size (X = 14.21, Y = 16.58 and Z = 11.86) were used for docking RVT with COV and SARS. The remaining values were set to default. Molecular dynamic simulation run was carried out with the aid of Amber 19 [34] software using the FF14SB force field [35]. The general Amber force field and restrained electrostatic potential were used in describing the atomic charges of RVT and TMC-310911. Leap variant present in Amber 19 was used for system neutralization and hydrogen atoms addition [36]. The system was solvated with an orthorhombic box of TIP3P water molecules surrounding all protein atoms at a distance of 9 Å [27]. System minimization was carried out first with a 2000 step minimization utilizing a restraint potential of 500 kcal/mol. Second, we used a 10,000-step full minimization process without restraint. Afterwards, the system was gradually heated at a temperature of 0–300 k at 50 ps. The system solutes are kept at a potential harmonic restraint of 10 kcal mol^-1^ Å^-2^ and collision frequency of 1.0 ps^-1^. Next, the equilibration of 500 ps was carried out. The temperature and pressure were kept constant at 300 k and 1 bar (isobaric-isothermal ensemble, constant temperature and pressure using Berendsen barostat). Each step of the simulation was run for 2 fs and an single-precision floating-point precision model was adopted. The simulations were kept at constant temperature and pressure, and Langevin thermostat at collision frequency of 1.0 ps^-2^. Six systems were set up for molecular dynamics (MD) simulations. These systems are unbound COV, COV bound to TMC-310911 (COV_TMC), COV bound to RVT (COV_RVT), unbound SARS, SARS bound to TMC-310911 (SARS_TMC) and SARS bound to RVT (SARS_RVT). PTRAJ variant of Amber 19 was adopted for further analysis which included root mean square deviation (RMSD) and radius of gyration (RoG) [37]. The data plots were then made with ORIGIN analytical tool and visualization was done using UCSF Chimera [38].

## Ritonavir & TMC-310911 binding free energy with COV & SARS

The molecular mechanics/Poisson–Boltzmann surface area (MM/PBSA) [39] was employed in the estimation of the binding strength in the COV_TMC, COV_RVT, SARS_TMC and SARS_RVT. MM/PBSA is described as an end-point energy estimation used in the calculation of binding mode of ligands and their corresponding protein target. MM/PBSA is described as:

(Eq. 1) $$\Delta {\text{G}}_{\text{bind}}\;={\text{ G}}_{\text{complex}}\;-\;({\text{G}}_{\text{receptor}}\;+{\text{ G}}_{\text{inhibitor}})$$

(Eq. 2) $$\Delta {\text{G}}_{\text{bind}}\;=\;\Delta {\text{G}}_{\text{gas}}\;+\;\Delta {\text{G}}_{\text{sol}}\;-\text{ T}\Delta \text{S}$$

(Eq. 3) $$\Delta {\text{G}}_{\text{gas}}\;=\;\Delta {\text{E}}_{\mathrm{int}}\;+\;\Delta {\text{E}}_{\text{ele}}\;+\;\Delta {\text{E}}_{\text{vdW}}$$

(Eq. 4) $$\Delta {\text{G}}_{\text{sol}}\;=\;\Delta {\text{G}}_{\text{ele},\text{sol}}{}_{\left(\text{GB}\right)}\;-\;\Delta {\text{G}}_{\text{np},\text{sol}}$$

(Eq. 5) $$\Delta {\text{G}}_{\text{np},\text{sol}}\;=\;\gamma \text{SASA }+\;\beta$$

ΔG_gas_ represents the total gas-phase energy calculated by intermolecular energy (ΔE_int_), electrostatic energy (ΔE_elel_) and van der Waals energy (ΔE_vdW_). ΔG_sol_ represents the solvation energy, TΔS represents entropy change. ΔG_ele,sol(GB)_ describes polar desolvation energy while ΔG_np,sol_ describes the nonpolar desolvation energy. The γ is the surface tension proportionality constant and is set to 0.0072 kcal mol^-1^ Å^-2^, β is a constant equal to 0 and SASA is the solvent-accessible surface area (Å^2^).

## Results

### Mechanism of inhibition of ritonavir & TMC-310911 on SARS-CoV-2 & SARS CoV main proteases

RVT and TMC-310911 (TMC) like any other HIV protease inhibitors work by inhibiting proteases found in the liver, intestine and other places [40,41]. The binding of RVT to the active site of HIV proteases prevents cleavage of the viral polyproteins subsequently leading to inactive and noninfectious viral components [15]. Similarly, TMC-310911 works following this pharmacokinetic and pharmacodynamic route [22]. Therefore, we explored the mechanism of inhibition and structural perturbative effect of RVT and TMC-3410911 binding to COV and SARS. MM/PBSA has been widely employed in the drug development and computer-aided drug designs space, it estimates the binding strength between an inhibitor and a protein [42,43]. COV_RVT, SARS_RVT, COV_TMC and SARS_TMC had binding free energy of -29.46, -32.34, -32.29 and -47.19 kcal/mol, respectively. Findings from this analysis revealed that the RVT and TMC-310911 had a favorable binding interaction with the COV and SARS (Table 1).

**Table 1.** Calculated binding free energy (kcal/mol) of the studied complexes.

Complexes	Energy kcal/mol (±SEM)						
	ΔE_vdW_	ΔE_ele_	ΔG_gas_	ΔG_ele,sol_	ΔG_nonpol,sol_	ΔG_sol_	ΔG_bind_
COV_RVT	-40.90 (±0.18)	-35.64 (±0.58)	-5.26 (±0.81)	-5.39 (±0.05)	-18.82 (±0.55)	-24.20 (±0.52)	-29.46 (±0.38)
SARS_RVT	-49.71 (±0.33)	-40.35 (±0.65)	-9.35 (±0.84)	-6.16 (±0.04)	-16.83 (±0.61)	-22.99 (±0.59)	-32.34 (±0.34)
COV_TMC	-42.41 (±0.39)	-41.21 (±0.16)	-18.5 (±1.44)	-5.25 (±0.04)	-15.80 (±0.01)	-23.21 (±1.19)	-32.29 (±0.05)
SARS_TMC	-55.38 (±0.52)	-14.95 (±1.04)	-20.49 (±1.38)	-6.51 (±0.06)	16.43 (±1.07)	15.77 (±1.04)	-47.19 (±0.58)

ΔE_ele_: Electrostatic energy; ΔE_vdW_: Van der Waals energy; ∆Gbind: Total binding energy; ΔG_ele,sol_: Desolvation energy; ΔG_gas_: Total gas-phase energy; ΔG_nonpol,sol_: Nonpolar desolvation energy; ΔG_sol_: Solvation energy; CoV: Coronavirus; COV_RVT: SARS-CoV-2 main protease bound to RVT; COV_TMC: SARS-CoV-2 main protease bound to TMC-310911; RVT: Ritonavir; SARS_RVT: SARS-CoV main protease bound to RVT; SARS_TMC: SARS-CoV main protease bound to TMC-310911; SEM: Standard error of the mean.

Furthermore, from Table 1, it is observed that the various free energy components facilitated this strong interaction. Most especially the electrostatic and van der Waals interactions observed in the gas phase of COV_TMC and SARS_TMC.

**Figure 1.**
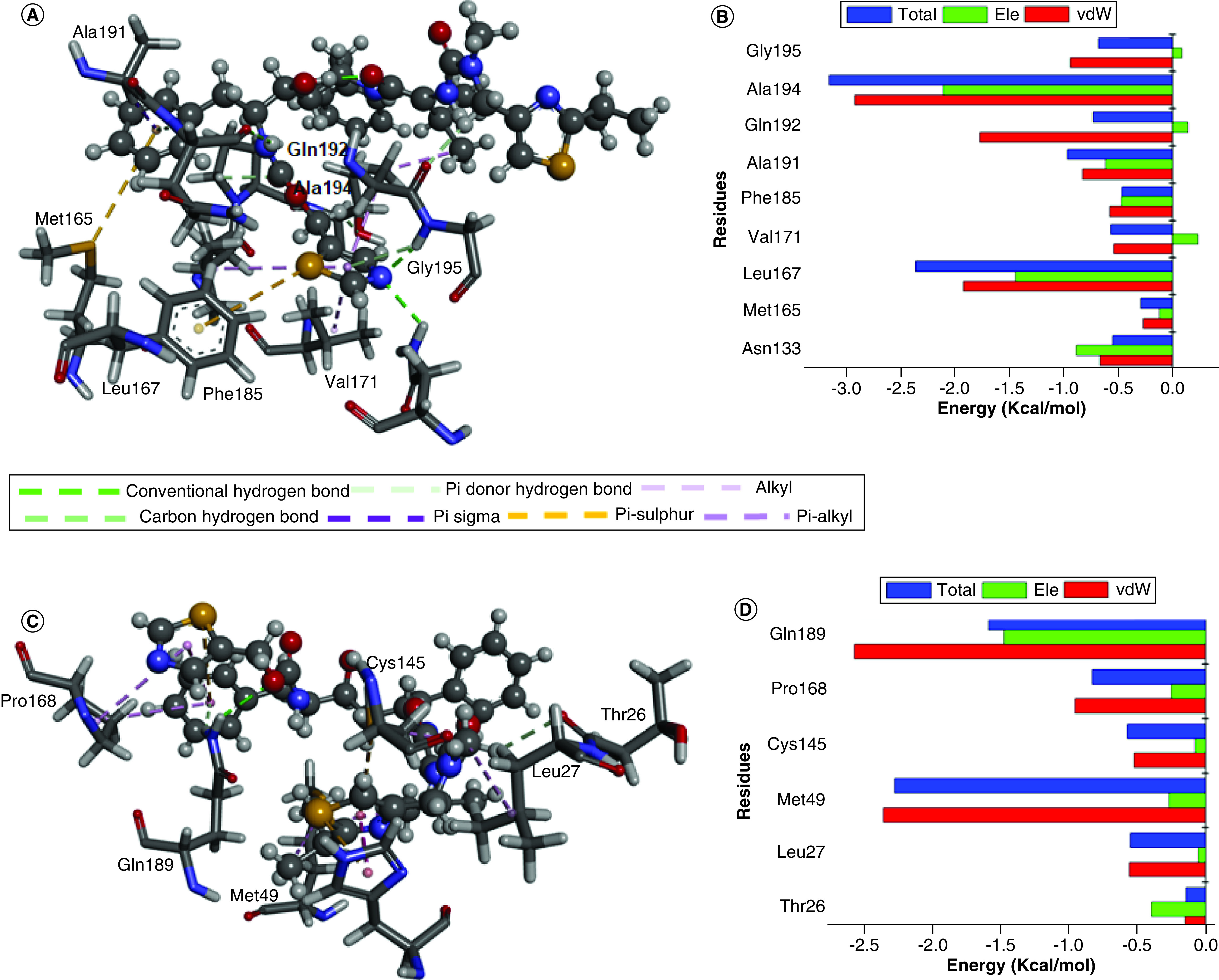
Per residue decomposition of interactions of ritonavir and SARS-CoV-2/SARS-CoV main proteases. **(A)** The most stable COV-ritonavir interaction, ritonavir is represented in ball and stick while interacting residues are represented in wires. **(B)** Per residue decomposition of important residues that contributed significantly to the binding. **(C)** The most stable SARS-ritonavir interaction, ritonavir is represented in ball and stick while interacting residues are represented in wires. **(D)** Per residue decomposition of important residues that contributed significantly to the binding. CoV: Coronavirus; COV-ritonavir: SARS-CoV-2 main protease bound to ritonavir; SARS-ritonavir: SARS-CoV main protease bound to ritonavir.

**Figure 2.**
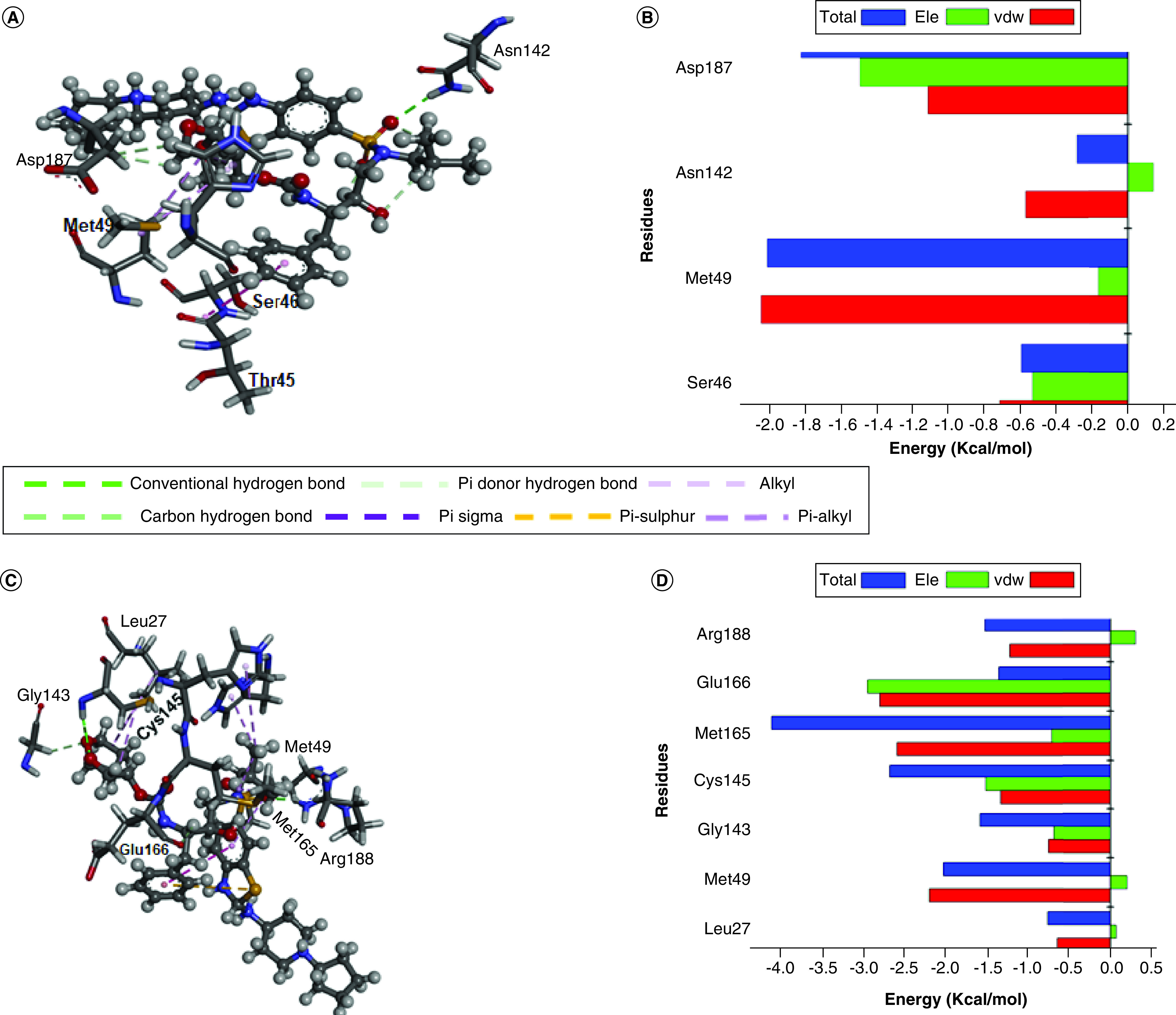
Per residue decomposition of interactions of TMC-310911 and SARS-CoV-2/SARS-CoV main proteases. **(A)** Illustration of the most stable COV_TMC interaction, TMC-310911 is represented in ball and stick while interacting residues are represented in wires. **(B)** Per residue decomposition of important residues that contributed significantly to the binding. **(C)** Illustration of the most stable SARS_TMC interaction, TMC-310911 is represented in ball and stick while interacting residues are represented in wires. **(D)** Per residue decomposition of important residues that contributed significantly to the binding. CoV: Coronavirus; COV_TMC: SARS-CoV-2 main protease bound to TMC-310911; SARS_TMC: SARS-CoV main protease bound to TMC-310911.

We used a representative snapshot and active site residue decomposition to further explore the time-wise bond interaction occurring between major residues in the active site of COV/SARS and TMC-310911/RVT. In the COV_RVT system, Asn133, Gly195 and Gln192 elicited hydrogen bond interactions, Phe185 formed π-sulphur interaction with the S atom present in RVT ring (Figure 1A). The decomposition of this interaction also showed that favorable energy was contributed by residues possessing energy contributions greater than -0.5 kcal/mol. Ala194, which formed a pi-alkyl bond with RVT, contributed the highest total, van der Waal, and electrostatic energies to the overall binding (Figure 1B). Decomposing the active site residues of the SARS_RVT system revealed that Met49 and Gln189 contributed the most to the overall binding of RVT to SARS. In comparison to the COV_RVT system, SARS_RVT had higher ΔG_bind_, this could be as a result of the high sigma bond formed by Pro168, Met49 and Cys145 (Figure 1C). Binding of TMC-310911 to COV and SARS demonstrated an overall favorable binding when compared with RVT binding. This is evident by the high energy decomposition of the individual active site residues. Asn142 and Asp187 elicited a high hydrogen bond, while Ser46, Thr45 and Met 49 formed a pi-alkyl bond with TMC-310911 (Figure 2).

**Figure 3.**
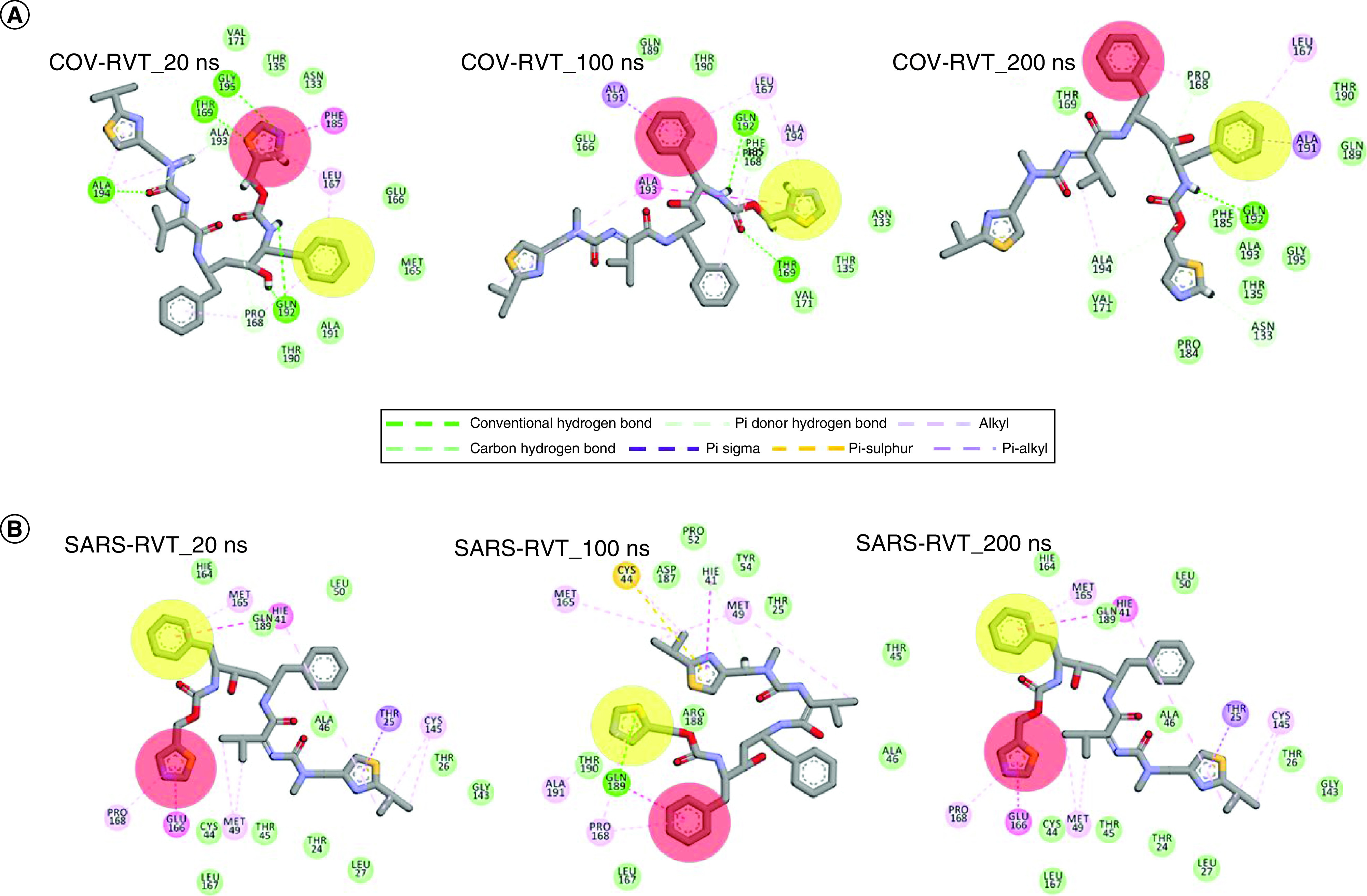
Time-based monitoring of COV-RVT (A) and SARS-RVT (B), highlighting the rings responsible for sequestering ritonavir into the active site of the proteins.

To further explore the possible mechanism of the action of RVT on SARS-CoV-2 main protease, we examined the interaction trend of two important rings in RVT in the course of the simulation run. Interestingly, these two rings formed strong interaction with residues embedded inside the active site. These interactions sequestered RVT deeper into the active site (Figure 3).

### COV & SARS perturbative effect upon ritonavir & TMC-310911 binding

**Figure 4.**
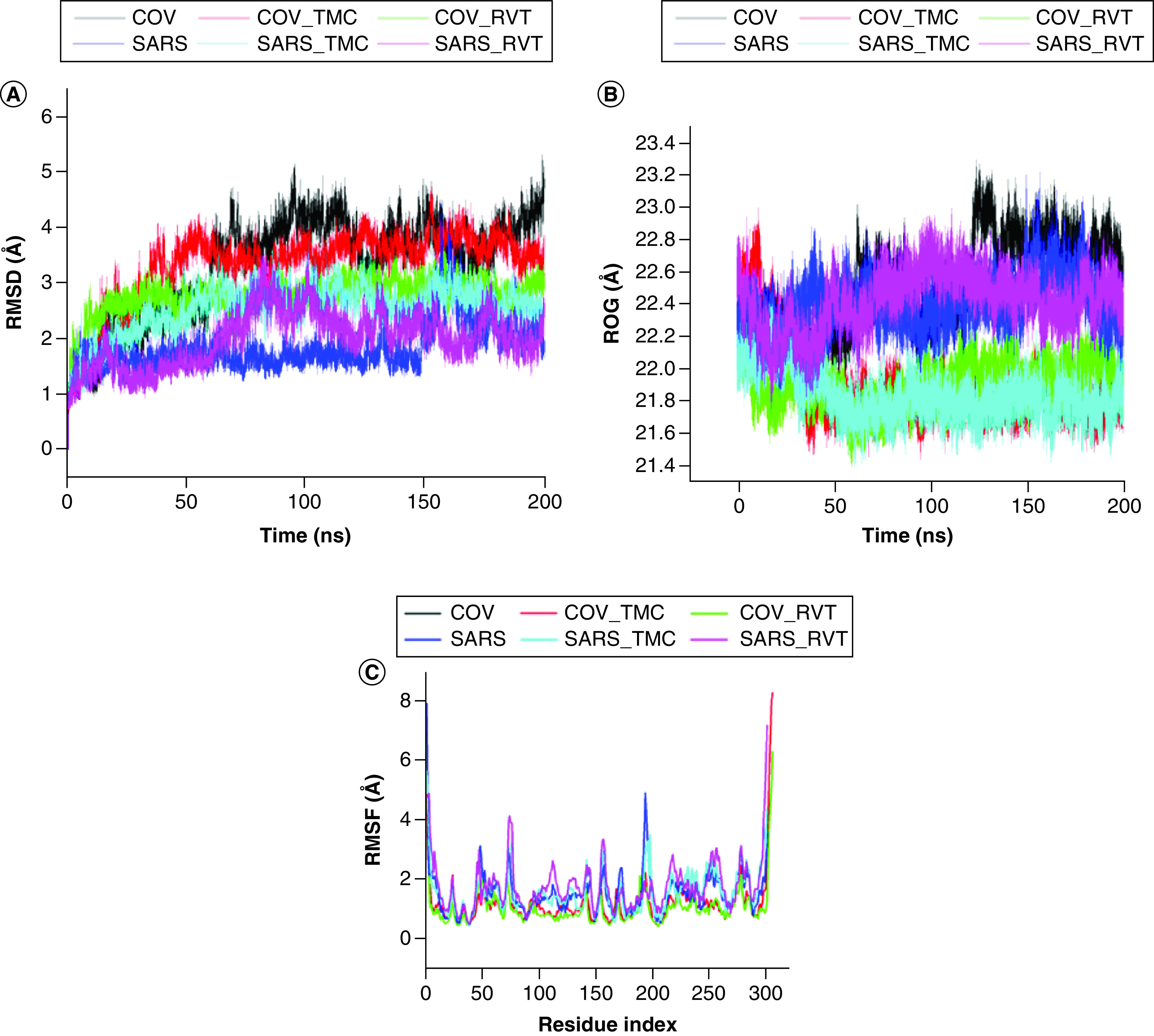
Structural and dynamic impact of ritonavir and TMC-310911 on SARS-CoV-2 and SARS-CoV main proteases. **(A)** Conformational analysis plot of the Cα RMSD plot demonstrating the stability of COV (black), COV_TMC (red), COV_RVT (green), SARS (purple), SARS_TMC (amber) and SARS_RVT (red). **(B)** Conformational analysis plot of the radius of gyration plot showing the movement of COV (black), COV_TMC (red), COV_RVT (green), SARS (purple), SARS_TMC (amber) and SARS_RVT (red). **(C)** RMSF plot of stability of COV (black), COV-TMC (red), COV-RVT (green), SARS (purple), SARS_TMC (amber) and SARS_RVT (red). COV: Unbound SARS-CoV-2 main protease; CoV: Coronavirus; COV_RVT: COV bound to RVT; COV_TMC: SARS-CoV-2 main protease bound to TMC-310911; RMSD: Root mean square deviation; RoG: Radius of gyration; RVT: Ritonavir; SARS: Unbound SARS-CoV main protease; SARS_RVT: SARS bound to RVT; SARS-TMC: SARS-CoV main protease bound to TMC-310911.

To understand the structural perturbation of COV and SARS upon RVT and TMC-310911 binding, we used RMSD and RoG to characterize the structural events in the proteins in the course of the simulation. In the course of the 200 ns simulation run, the six systems attained structural stability early in the simulation run with COV, COV_TMC, COV_RVT, SARS, SARS_TMC and SARS_RVT having an average RMSD value of 3.43, 3.34, 2.77, 1.85, 2.56 and 2.00Å, respectively (Figure 4A). The average RMSF value of COV, COV_TMC, COV_RVT, SARS, SARS_TMC and SARS_RVT are 1.19, 1.19, 1.06, 1.50, 1.57 and 1.77Å, respectively. Likewise, COV, COV_TMC, COV_RVT, SARS, SARS_TMC and SARS_RVT had average RoG values of 22.53, 21.89, 21.92, 22.41, 21.85 and 22.42Å, respectively (Figure 4B).

## Discussion

Sequence similarity is described as a measure of the empirical relationship that exists between sequences, it establishes the propensity of sequences evolving from a common ancestor. Joshi *et al.* have demonstrated that COV has higher sequence similarity with SARS when compared with MERS-CoV main protease [44]. In this study, RVT and TMC-310911 were repurposed to bind with COV and SARS. Although there are conflicting results on the therapeutic effectiveness of RVT and TMC-310911 in the treatment of COVID-19. For instance, in a clinical trial study, the combination of lopinavir and RVT was investigated in a controlled trial study, patients with COVID-19 were administered either lopinavir-RVT 400/100 mg orally twice daily plus standard of care or standard of care alone, there was no therapeutic benefit observed upon administration [20]. RMSD is a commonly used quantitative parameter employed to estimate the similarity between two superimposed structures. RMSD can be computed for different components of a biomolecule. In MD simulation, the RMSD is often calculated for the Cα of the entire protein structure, for example, those found in the loop, active site and perhaps transmembrane helices. Many types of researches have used RMSD as a measure of protein stability and equilibration. RMSF is defined as the measure of the atomic displacement of a single or a group of atoms relative to the starting or reference structures, averaged over the number of atoms [45]. RoG is a function used to define the distribution of atoms of a protein around its axis. The most significant parameter used in the prediction of protein compactness is RoG [46]. Insight from RMSD, RMSF and RoG revealed the dynamical events occurring upon TMC-310911 and RVT binding to COV and SARS. The binding score derived from docking of RVT and TMC-310911 to COV and SARS revealed that the COV-RVT had a binding score of -7.09 kcal/mol, SARS_RVT, COV_TMC and SARS_TMC had -8.12, -8.00 and -9.09 kcal/mol, respectively. Similarly, an increasing trend was observed in the binding free energy of the systems as computed by MM/PBSA. COV_RVT, SARS_RVT, COV_TMC and SARS_TMC had binding free energy of -29.46, -32.34, -32.29 and -47.19 kcal/mol, respectively. Nukoolkarn *et al.* used MD simulation to explore the binding of RVT to SARS-CoV protease, from their study, the total binding energy of -45.3 kcal/mol was observed [47]. Upon binding of RVT to SARS-CoV-2 main protease, the structural compactness of the COV_TMC, COV_RVT system were lesser than that of the COV system, similar trend was observed in the SARS_TMC and SARS_RVT systems.

## Conclusion

Presently, there is no available clinical antiviral compound (or drug) with therapeutic evidence that cures COVID-19 and several strategies are being considered to treat this disease, including repurposing drugs that are active against SARS-CoV and MERS-CoV. Other strategies such as inhibitors of viral and host protease, host-directed therapies and the use of antibodies are being currently explored. In this study, we explored the inhibitory effect of RVT and TMC-310911 on COV and SARS. Results from this study revealed that RVT and TMC-310911 had strong binding interaction with COV and SARS. Two rings were found to be crucial to the binding of RVT to COV and SARS. These two rings could be further explored in the development of inhibitors specifically tailored to target SARS-CoV-2 proteases.

## Future perspective

We employed integrated computational algorithms and force field protein–ligand dynamics calculations to repurpose and explore the possible mechanism of inhibition of RVT and TMC-310911 upon binding to COV and SARS. Findings from this study can facilitate a new frontier in structure-based design of highly effective and tailored inhibitors of SARS-CoV-2 main protease in the treatment of CoV infections.

**Summary points**

- Upon ritonavir (RVT) binding to SARS coronavirus 2 (SARS-CoV-2) main protease (COV), Asn133, Gly195 and Gln192 elicited hydrogen bond interactions, while Phe185 formed π-sulphur interaction with the S atom present in the RVT ring.
- RVT and TMC-310911 had strong interactions with the proteases, and these high interactions are facilitated by some significant residues found in their active site.
- Despite having similar residues in their active sites, COV elicited ligand interactions with dissimilar residues as SARS-CoV protease.
- Two rings were found to be crucial to the binding of RVT to COV and SARS-CoV main proteases.
